# Effect of Endodontic Sealers on the Bond Strength of Glass Fibre Posts: A Systematic Review

**DOI:** 10.1111/aej.70066

**Published:** 2026-03-11

**Authors:** Thiago Bessa Marconato Antunes, Juliana D. Bronzato, Vanessa Gallego Arias Pecorari, Jennifer Santos Pereira, Talita Tartari, Adriana de Jesus Soares, Brenda P. F. A. Gomes, Marina Angélica Marciano

**Affiliations:** ^1^ Department of Restorative Dentistry, Piracicaba Dental School State University of Campinas‐UNICAMP Piracicaba Sao Paulo Brazil; ^2^ Department of Social Dentistry, Piracicaba Dental School State University of Campinas‐UNICAMP Piracicaba Sao Paulo Brazil

**Keywords:** fibreglass post, push‐out, resin cements, root canal filling materials, systematic review

## Abstract

This systematic review evaluated the influence of resin‐based, eugenol‐based and bioceramic endodontic sealers on the bond strength of glass fibre posts cemented with resin cements. Searches were conducted in PubMed, Scopus, Web of Science, Cochrane Library, Google Scholar and Embase, including in vitro studies using human or bovine teeth restored with fibre posts. Risk of bias was assessed using the QUIN Tool. Thirty three studies met the eligibility criteria. Most evidence indicates that resin‐based sealers promote higher bond strength values than eugenol‐based sealers, whereas findings for bioceramic sealers were inconsistent. Only a minority of studies showed a low risk of bias, while most presented moderate methodological limitations. Substantial heterogeneity was also observed, including differences in irrigation protocols, sealer types, adhesive strategies and post‐cementation intervals. Within the limitations of the available evidence, resin‐based sealers appear to be the most favourable choice for clinical fibre post cementation.

**PROSPERO Registration Number:** CRD42023467018.

## 1 Introduction

Endodontically treated teeth that require prosthetic rehabilitation, particularly those with extensive coronal destruction and loss of dental structure, often demand intraradicular posts to provide adequate support for the final restoration [1]. Fibreglass posts present biomechanical advantages over metallic posts, mainly due to their modulus of elasticity (20 GPa), which is similar to that of dentine (18 GPa), reducing the risk of root fractures [2]. In addition, fibreglass posts are corrosion‐resistant, do not require laboratory processing, can be adhesively cemented in a single clinical session, provide favourable aesthetics and exhibit translucency that facilitates light transmission and polymerisation of resin cements [2, 3, 4, 5].

Several factors influence the bond strength between dentine and resin cement during the cementation of fibreglass posts, and the root canal sealer is among the most relevant [6]. The effects of eugenol‐based sealers are well documented, with numerous studies reporting reduced bond strength values when these materials are used [7, 8, 9, 10]. This decrease has been attributed to the ability of eugenol to interfere with the polymerisation of resin‐based materials [8]. Although the exact mechanism remains unclear, one systematic review suggests that residual eugenol entrapped within the smear layer may release hydroxyl ions capable of neutralising resin monomer free radicals, thereby reducing polymerisation efficiency [9]. However, this mechanism becomes less plausible when appropriate endodontic protocols are followed, such as the final EDTA rinse to remove the smear layer [11]. Another widely discussed hypothesis is that residual eugenol or its by‐products may remain adsorbed onto dentine or penetrate into dentinal tubules, releasing phenolic radicals that inhibit polymerisation and compromise adhesion [7, 10].

When root canals are filled with resin‐based sealers, the bond strength values are generally high due to their chemical similarity with resin cements, which favours interfacial compatibility and polymerisation [10]. In contrast to this predictable behaviour, several studies have consistently reported lower bond strength values for calcium silicate–based sealers when compared with resin‐based sealers [12, 13, 14, 15, 16, 17, 18]. According to these authors, this effect may be related to the strong chemical interaction between bioceramic sealers and dentine, as well as the formation of a highly alkaline environment during the hydration process. The release of calcium hydroxide and the subsequent alkalinization promote mineral precipitation and intratubular crystal formation, which can modify the dentine surface and interfere with resin infiltration. Consequently, instead of bonding directly to dentine, the resin cement may adhere to a layer of mineralized or sealer‐derived material, potentially altering the mechanical performance of the bonded interface. The influence of calcium silicate–based sealers on bond strength, however, remains unclear, as the literature reports contradictory results regarding their interaction with resin cements [12, 13, 15, 16, 17, 19, 20, 21, 22].

From the best of the author's knowledge, this is the first systematic review to investigate if calcium silicate–based root canal sealers affect the bond strength of fibreglass posts compared with eugenol‐based and resin‐based sealers, including both methacrylate and epoxy resin formulations. Therefore, the aim of this study was to comprehensively evaluate, through a systematic review, the influence of these different types of endodontic sealers on the bond strength between fibreglass posts and root canal dentine.

## 2 Methods

### 2.1 Study Design

This systematic review was conducted in accordance with the PRISMA (Preferred Reporting Items for Systematic Reviews and Meta‐analyses) statement guidelines [23, 24] and registered at PROSPERO under protocol number CRD42023467018.

### 2.2 Pecos

The research question guiding this review was: ‘Does the type of endodontic sealer influence the bond strength of fiberglass posts cemented with resin‐based cement?’ This research question was constructed based on the PECOS strategy, namely: Population (bovine or extracted human endodontically treated teeth/roots with intracanal fiberglass post); Exposure (endodontic treatment and fiberglass post cementation); Comparison (different endodontic sealers); Outcome (bond strength); Study design (in vitro).

### 2.3 Search Strategy

In order to identify studies to be included in this review, the search strategy covered six electronic databases (i.e., PubMed, Embase, Scopus, Web of Science, Google Scholar and the Cochrane Library) from their start dates (1978) to October 7, 2025. The search strategy was developed using a combination of free‐text terms and MeSH (Medical Subject Headings) descriptors related to tooth type, glass fibre posts, endodontic sealers and bond strength testing methods.

The search terms included variations referring to human or bovine teeth, different nomenclature for glass fibre posts, and descriptors associated with endodontic filling materials. In addition, terms related to mechanical bond strength evaluation (push‐out, pull‐out, microtensile) were combined with in vitro or laboratory studies terms. These components were structured into four main search blocks, which were then combined using Boolean operators (1 AND 2 AND 3 AND 4) to produce the final search set. This strategy was applied to identify relevant studies indexed in major scientific databases Table 1.

**TABLE 1:** Search strategy.

Search terms (free‐text terms) identified in the title, abstract, or keywords	Concept
1 (‘bovine tooth’ OR ‘bovine teeth’ OR ‘human tooth’ OR ‘human teeth’ OR ‘bovine root’ OR ‘human root’ OR ‘tooth roots’ [MeSH])	Tooth type (human or bovine)
2 (‘glass fibre post’ OR ‘glass fibre post’ OR ‘fibreglass post’ OR ‘fibreglass post’ OR ‘glassfiber post’ OR ‘glassfibre post’ OR ‘fibreglass pin’ OR ‘glass fibre pin’ OR ‘glass fibre pin’ OR ‘fibreglass’ [MeSH] OR ‘fibreglass’ [MeSH] OR ‘Post Technique’ [MeSH])	Glass fibre posts (terminological variants and MeSH terms)
3 (‘endodontic sealer’ OR ‘root canal filling’ OR ‘endodontic cement’ OR ‘Root Canal Filling Materials’ [MeSH])	Endodontic sealers/root canal filling materials
4 (‘bond strength’ OR ‘push‐out’ OR ‘pull‐out’ OR ‘microtensile’) AND (‘in vitro’ [MeSH] OR ‘laboratory studies’ OR ‘laboratory study’)	Bond strength testing methods
1 AND 2 AND 3 AND 4	Final combination of descriptors

The reference lists of included studies were also hand‐searched to identify additional relevant studies. The search was performed by one of the authors with experience in literature search for systematic review.

### 2.4 Eligibility Criteria

All references were tabulated using EndNote Online, and duplicates (articles retrieved from more than one database) were removed. During the screening process, titles and abstracts were carefully reviewed to remove studies that were out of scope. When the title or abstract was unclear, the full text was retrieved and assessed prior to exclusion. Subsequently, the full texts of the selected studies were evaluated according to the following inclusion criteria: in vitro studies using bovine or extracted human teeth cemented with fibreglass post and resin‐based cement after root canal filling evaluated for bond strength to root dentine.

The exclusion criteria were: case reports, case series, conference abstracts, opinion letters, clinical studies, reviews, book chapters, full text not available, articles not written in English, lack of quantitative data, studies using sealers not included in the review and studies that did not compare two or more endodontic sealers (i.e., calcium silicate–based vs. resin‐based; calcium silicate–based vs. eugenol‐based; or eugenol‐based vs. resin‐based). Additionally, any in vivo or ex vivo animal experiments were excluded, as the review focused solely on in vitro assessments using extracted teeth.

All analyses were performed by two independent reviewers (TBMA, JDB), and in cases of disagreement, a consensus was reached through discussion with a third reviewer (MM). When relevant information was missing, the corresponding authors of the articles were contacted for clarification.

### 2.5 Data Collection

After selection of the studies, the following data were collected: authors, year of publication, number of samples in each group, mean value and standard deviation of bond strength, type of irrigant used in the study, type of resin cement used for cementing the posts, type of mechanical test (push‐out or pull‐out), type and group of teeth and the obturation technique used. An author (TBMA) collected all this data from the included studies, and a second author (JDB) checked it. Any disagreement was consensually solved and, if not, a third author (MM) was consulted.

### 2.6 Risk of Bias

The quality assessment of the included studies was performed independently by two authors (TBMA, JDB) by using the Quality Assessment Tool for In Vitro Studies (QUIN) [25]. Each criterion was evaluated to assess the risk of bias of in vitro studies and was marked under the categories of adequately specified, inadequately specified, not specified, or not applicable. The reviewers scored each of the 12 criteria as adequately specified = 2 points, inadequately specified = 1 point, not specified = 0 point and not applicable = exclude criteria from calculation. The scores were then added to obtain a total score for a particular in vitro study. The scores obtained were used to grade the in vitro study as high, medium, or low risk (> 70% = low risk of bias, 50% to 70% = medium risk of bias and < 50% = high risk of bias) by using the following formula:

Final score = (Total score × 100)/(2 × number of criteria applicable).

### 2.7 Data Analysis

A meta‐analysis was not performed in this study due to the considerable methodological heterogeneity among the included articles. Differences in study designs, types of endodontic sealers evaluated, specimen preparation methods, bond strength testing protocols and outcome criteria prevented the quantitative combination of data in a valid and reliable manner. Therefore, we opted to conduct only a qualitative synthesis to ensure the integrity and robustness of the interpretation of the available evidence.

## 3 Results

**FIGURE 1:**
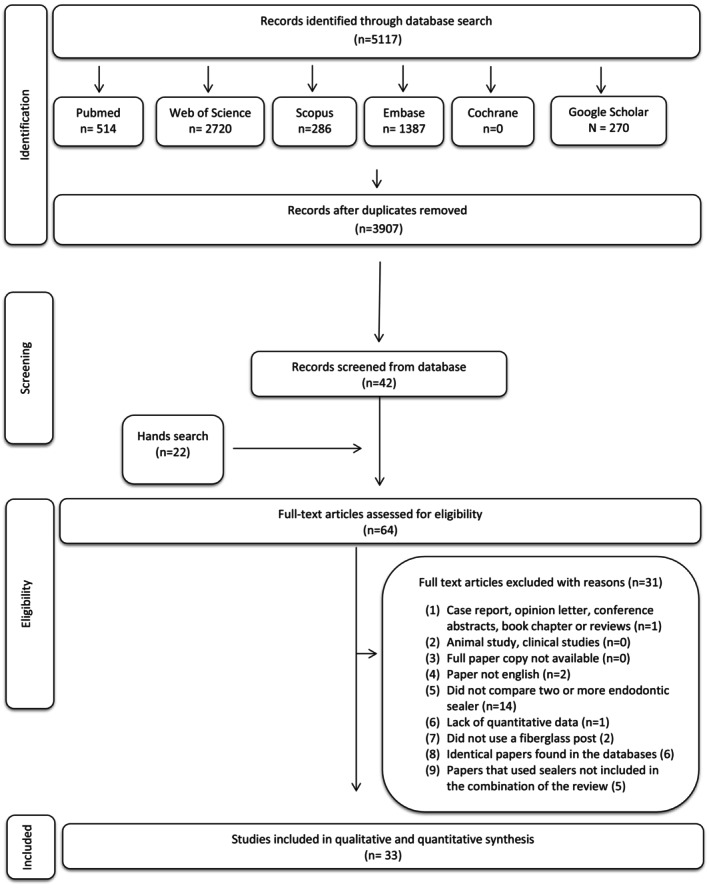
PRISMA flow diagram representing the study design process of this systematic review.

Figure 1 summarises the study selection process. After removal of duplicates, 5117 records were identified through database searching. Following the eligibility criteria, 64 full‐text articles were assessed and 33 studies were finally included in the qualitative analysis (Table 2). Among the included studies, 28 used human teeth, whereas 5 used bovine teeth. Regarding the bond strength assessment, 29 studies employed the push‐out test and 4 studies used the pull‐out test.

**TABLE 2:** Descriptive data of included studies in the sistematic review.

Authors	Endodontic sealer	Simple size	Irrigating solutions	Type of resin cement	Adhesive system	Test	Substate	Filling technique	Post cementation time
Kurtz et al. [26]	Roth's 801 (Roth International Ltd)	2	NaOCl 5.25%	Post cement Hi‐X (Bisco)	One Step (Bisco)	Push‐out (2–3 mm)	Human single‐rooted maxillary central incisors and canines	Vertical warm condensation	1 week
	AH 26 (Dentsply)			Parapost cement (Coltene)	Parapost cement conditioner (Coltene)				
Baldissara et al. [27]	Topseal (Dentsply)	10	NaOCl 5%	Bisfil 2B (Bisco)	Allbond2 (Bisco)	Push‐out (1 mm)	Single‐rooted human teeth	Vertical condensation	2 weeks
	Pulp canal Sealer (Kerr)								
Menezes et al. [28]	Sealer 26 (Dentsply)	6	NaOCl 1%	RelyX ARC (3 M ESPE)	Adper scotchbond multi purpose (3 M ESPE)	Push‐out (1 mm)	Bovine teeth	Lateral compaction	Immediate and 1 week
	Endofill (Dentsply)								
Demiryürek et al. [29]	AH plus (Dentsply)	12	NaOCl 0.5%	Panavia F 2.0 (Kuraray)		Push‐out (0,6 mm)	Single‐rooted human maxillary central incisors teeth	Cold lateral compaction	1 week
	Endofill (Dentsply)								
Cecchin et al. [30]	EndoREZ (Ultradent)	10	NaOCl 2.5%	RelyX unicem (3 M ESPE)		Push‐out (1 mm)	Single rooted human teeth	Cold lateral compaction	1 week
	Endofill (Dentsply)								
Cecchin et al. [31]	AH plus (Dentsply)	10	NaOCl 2.5%	RelyX Unicem (3 M ESPE)		Push‐out (1 mm)	Human maxillary single‐rooted canine teeth	Cold lateral compaction	1 week
	Endomethasone (Septodont)								
Aggarwal et al. [8]	AH Plus (Dentsply)	10	NaOCl 5.25%	Paracore (Coltene)	Parabond non‐rinse conditioner	Push‐out (1,2 mm)	Human uniradicular mandibular premolar teeth	Lateral compaction	1 week
	Zinc oxide eugenol sealer								
Aleisa et al. [7]	AH 26 (Dentsply)	15	NaOCl 5.25%	RelyX unicem (3 M ESPE)		Pull‐out	Single rooted human teeth	Lateral condensation	24 h
	Endofil (Dentsply)								
Gomes et al. [32]	N‐Rickert (Biodinâmica)	5	NaOCl 0.5%	Bistite II DC (J Morita)	Primer 1A and 1B (Bistite)	Pull‐out	Single‐rooted bovine incisor teeth	Lateral condensation	72 h
	Sealer 26 (Dentsply)								
Gündoğar & Gençoğlu [33]	AH plus (Dentsply)	10	NaOCl 5.25%	Duo‐link (Bisco)		Push‐out (3 mm)	Human maxillary central incisor teeth	Lateral compaction	1 week
	PulpCanal Sealer (Kerr)								
Özcan et al. [19]	AH plus Jet (Dentsply)	12	NaoCl 2.5%	Clearfil SA (Kuraray)		Push‐out (1 mm)	Human maxillary incisor teeth	Cold lateral condensation	1 week
	Endofill (Dentsply)								
	iRoot SP (Innovative BioCremix Inc)								
Aleisa et al. [34]	AH 26 (Dentsply)	18	NaOCl 5.25%	Multicore flow (Ivoclar)	AdheSE (Ivoclar Vivadent)	Pull‐out	Single‐rooted human permanent mandibular first premolar teeth	Lateral condensation	1 week
	Endofill (Dentsply)								
Rosa et al. [35]	AH Plus (Dentsply)	20	NaOCl 1%	AllCem (FGM)	Scotchbond multipurpose (3 M ESPE)	Push‐out (2 mm)	Single‐rooted bovine teeth	Lateral compaction	Immediate and 15 days
	Endofill (Dentsply)								
Mosharraf and Zare [36]	AH 26 (Dentsply)	10	NaOCl 5.25%	Panavia F 2.0 (Kuraray)		Push‐out (3 mm)	Human mandibular first premolar teeth	Vertical condensation	1 week
	Endofill (Dentsply)								
Al‐Dwairi et al. [37]	AH26 (Dentsply)	10	NaOCl 5.25%	RelyX unicem (3 M ESPE)		Push‐out	Single‐rooted human mandibular premolar teeth	Lateral condensation	1 week
	Endofill (Dentsply)			Variolink II (Ivoclar)					
				ParaCore (Coltene)					
Sukuroglu [38]	AH plus (Dentsply)	8	NaOCl 5.25%	Variolink II (Ivoclar)	Heliobond (Ivoclar)	Push‐out (1 mm)	Single‐rooted mandibular premolar human teeth	Cold lateral condensation	1 week
	Sealite (Septodont)								
	iRoot SP (Innovative BioCremix Inc)								
Lima et al. [39]	AH plus (Dentsply)	10	NaOCl 1%	RelyX U100 (3 M ESPE)		Push‐out (1 mm)	Maxillary human canine teeth	Lateral compaction	Three times the setting time of the sealers
	Endofill (Dentsply)								
Reyhani et al. [40]	AH plus (Dentsply)	18	NaOCl 2.5%	Clearfil SA (Kuraray)		Push‐out (1 mm)	Human single‐rooted maxillary incisors teeth	Cold lateral compaction	1 week
	Dorifill (Dorident)								
Dibaji et al. [12]	AH plus (Dentsply)	14	NaOCl 2.5%	Panavia F 2.0 (Kuraray)		Push‐out (1 mm)	Single canal human premolar teeth	Cold lateral compaction	1 week
	Dorifill (Dorident)								
Bohrer et al. [10]	AH plus (Dentsply)	15	NaOCl 2.5%	RelyX U200 (3 M ESPE)		Push‐out (1,5 mm)	Bovine incisor teeth	Cold lateral condensation	24 h, 6 months and 12 months
	Endofill (Dentsply)			Multilink (Ivoclar)					
Ruiz et al. [41]	Endofill (Dentsply)	7	NaOCl 1%	RelyX U200 (3 M ESPE)		Push‐out (1 mm)	Human maxillary canine teeth	Vertical warm condensation	1 week and 6 months
	Sealer 26 (Dentsply)			Variolink II (Ivoclar)					
Vilas‐Boas et al. [13]	Endofill (Dentsply)	12	NaOCl 2.5%	RelyX ARC (3 M ESPE)	Adapter scotchbond multi purpose (3 M ESPE)	Push‐out (1,5 mm)	Single‐rooted mandibular premolar human teeth	Vertical compaction	Immediate and 1 week
	EndoSequence BC Sealer (Brasseler)								
	AH Plus (Dentsply)								
Nascimento et al. [42]	AH Plus (Dentsply)	10	Saline solution	RelyX U200 (3 M ESPE)	Adapter scotchbond multi purpose (3 M ESPE)	Push‐out (1 mm)	Humansingle‐rooted mandibular first premolars	Cold lateral condensation	15 days
	Endofill (Dentsply)			RelyX ARC (3 M ESPE)					
Bengoa et al. [14]	Bio‐C Sealer (Angelus)	20	NaOCl 2.5%	RelyX U200 (3 M ESPE)		Push‐out (1 mm)	Upper and lower human premolar	Vertical compaction	1 week
	AH Plus (Dentsply)								
Alsubait et al. [16]	AH plus (Dentsply)	15	NaoCl 2.5%	Rely X unicem (3 M ESPE)		Pull‐out	Human maxillary central incisors	Warm vertical compaction	Immediate and 1 week
	Endosequence BC sealer HiFlow (Brasseler)								
Bohrer et al. [43]	AH plus (Dentsply)	24	NaOCl 2.5%	RelyX U200 (3 M ESPE)		Push‐out (1,5 mm)	Bovine incisor teeth	Cold lateral condensation	24 h and 30 months
	Endofill (Dentsply)								
Soares et al. [15]	AH plus (Dentsply)	12	NaOCl 2.5%	Panavia F 2.0 (Kuraray)		Push‐out (1 mm)	Human canine teeth	Lateral condensation	Not mentioned
	Endofill (Dentsply)								
	EndoSequence BC Sealer (Brasseler)								
	Sealer plus (MK Life)								
dos Santos et al. [44]	AH plus (Dentsply)	13	NaOCl 1%	Light core (Bisco)	Optibond solo Plus (Kerr)	Push‐out (1 mm)	Human maxillary central incisor teeth	Vertical compaction	72 h
	Endofill (Dentsply)								
Yuanli et al. [21]	AH plus (Dentsply)	8	Chlorhexidine 2%	RelyX U200 (3 M ESPE)		Push‐out (1 mm)	Single‐rooted human premolar teeth	Warm vertical compaction	Immediate and 1 week
	iRoot SP (Innovative BioCremix Inc)								
Nesello et al. [17]	AH plus (Dentsply)	10	NaoCl 2.5%	RelyX U200 (3 M ESPE)		Push‐out (2 mm)	Single‐rooted premolar human teeth	Vertical compaction	1 week
	Bio‐C Sealer (Angelus)			RelyX ARC (3 M ESPE)	Adapter scotchbond multi purpose (3 M ESPE)				
	Sealer Plus BC (MK life)								
Pinto et al. [22]	AH plus (Dentsply)	15	NaoCl 2.5%	Panavia F 2.0 (Kuraray)	ED PRIMER II (Kuraray)	Push‐out (1 mm)	Single‐rooted human premolars	Vertical compaction	48 h
	Endofill (Dentsply)								
	Bio‐C sealer (Angelus)								
Porto et al. [18]	AH plus (Dentsply)	10	NaoCl 2.5%	RelyX U200 (3 M ESPE)	Adapter scotchbond multi purpose (3 M ESPE)	Push‐out (2 mm)	Human maxillary central incisors	Vertical compaction	1 week
	Bio‐C Sealer (Angelus)			RelyX ARC (3 M ESPE)					
Antunes et al. [20]	AH plus (Dentsply)	12	NaoCl 2.5%	RelyX ARC (3 M ESPE)	Adapter scotchbond multi purpose (3 M ESPE)	Push‐out (1 mm)	Single‐rooted human maxillary incisors	Vertical compaction	28 days
	Endosequence BC sealer HiFlow (Brasseler)								

Table 2 presents all authors of the selected studies along with the respective sample size, the irrigant solution used in the chemomechanical preparation of the root canals, as well as the sealer, adhesive system, and resin cement used for post cementation. Additionally, it includes the type of mechanical test, substrate type, obturation technique, and post cementation time. Only Soares et al. [15] did not report the post cementation time.

### 3.1 Risk of bias

The risk of bias is shown in Table 3. Only seven studies presented a low risk of bias [15, 16, 17, 18, 20, 30, 41], 20 studies presented a moderate risk of bias [8, 12, 13, 14, 18, 21, 22, 27, 28, 29, 31, 34, 35, 36, 37, 38, 39, 40, 42, 44], and six studies presented a high risk of bias [7, 10, 26, 32, 33, 43]. The most frequent reasons for low scores were the absence of sample size calculation (Q2), lack of randomization or inadequate explanation of the method used (Q7), and the absence of blinding of operators or outcome assessors (Q10). Additionally, many studies did not describe the outcome assessor (Q9) or the sampling technique (Q3) or provided insufficient detail regarding the operator training and role (Q6). These methodological weaknesses contributed to the assignment of 1 or 0 points, thereby influencing the overall risk of bias classification.

**TABLE 3:** Risk of bias and scoring sheet for QUIN tool.

Study	Q1	Q2	Q3	Q4	Q5	Q6	Q7	Q8	Q9	Q10	Q11	Q12	Total (% score yes)	Risk of bias
Kurtz et al. [26]	2	0	1	1	1	0	1	0	0	0	2	2	41.7	Hight
Baldissara et al. [27]	2	0	1	1	2	0	1	2	0	0	1	2	50	Medium
Menezes et al. [28]	2	0	1	2	2	0	2	2	0	0	1	2	58.33	Medium
Demiryürek et al. [29]	2	0	0	2	2	0	1	2	0	0	2	2	54.16	Medium
Cecchin et al. [31]	2	2	1	2	2	2	2	2	0	0	1	2	75	Low
Cecchin et al. [30]	2	0	1	2	2	2	1	2	0	0	1	2	62.5	Medium
Aggarwal et al. [8]	2	0	1	2	2	0	0	2	0	0	2	2	54.16	Medium
Aleisa et al. [7]	2	0	0	1	2	0	0	2	0	0	2	2	45.83	High
Gomes et al. [31]	2	0	0	1	1	0	1	2	0	0	2	1	41.66	High
Gündoğar and Gençoğlu [32]	2	1	0	1	1	0	1	2	0	0	1	2	45.83	High
Özcan et al. [19]	2	0	2	2	2	2	1	2	0	0	1	2	66.7	Medium
Aleisa et al. [34]	2	0	0	2	2	0	1	2	0	0	2	2	54.16	Medium
Rosa et al. [35]	2	0	1	1	2	2	1	2	0	0	1	2	58.33	Medium
Mosharraf and Zare [36]	2	0	1	1	2	1	1	2	0	0	2	2	58.33	Medium
Al‐Dwairi et al. [37]	2	0	1	1	2	0	1	2	0	0	1	2	50	Medium
Sukuroglu [38]	2	0	2	2	2	1	1	2	0	0	2	2	66.7	Medium
Lima et al. [39]	2	0	2	1	2	0	0	2	0	0	2	2	54.16	Medium
Reyhani et al. [40]	2	0	1	2	2	1	1	2	0	0	2	2	62.5	Medium
Dibaji et al. [12]	2	0	1	2	2	0	1	2	0	0	1	2	54.16	Medium
Bohrer et al. [10]	2	0	0	1	2	0	1	2	0	0	1	2	45.83	High
Ruiz et al. [45]	2	0	2	1	2	2	1	2	1	0	2	2	70.83	Low
Vilas‐Boas et al. [13]	2	0	1	2	2	2	1	2	0	0	2	2	66.7	Medium
Nascimento et al. [42]	2	0	2	2	2	0	1	2	0	0	2	2	62.5	Medium
Alsubait et al. [16]	2	0	1	2	2	2	1	2	1	0	2	2	70.84	Low
Bengoa et al. [14]	2	0	1	2	2	0	2	2	0	0	2	2	62.5	Medium
Bohrer et al. [43]	2	0	0	1	2	0	1	2	0	0	1	2	45.83	High
Soares et al. [15]	2	0	2	2	2	2	1	2	0	0	2	2	70.84	Low
dos Santos et al. [44]	2	2	2	1	2	0	1	2	0	0	2	2	66.7	Medium
Yuanli et al. [21]	2	0	2	1	2	2	1	2	0	0	2	2	66.7	Medium
Nesello et al. [17]	2	2	2	1	2	2	1	2	1	0	2	2	79.16	Low
Pinto et al. [22]	2	2	1	1	2	0	1	2	0	0	2	2	62.50	Medium
Porto et al. [18]	2	2	2	1	2	2	1	2	0	0	2	2	75	Low
Antunes et al. [20]	2	2	2	2	2	0	1	2	0	0	2	2	70.83	Low

*Note:* Q1 (clearly stated aims/objectives); Q2 (detailed explanation of sample size calculation); Q3 (detailed explanation of sampling technique); Q4 (details of comparison group); Q5 (detailed explanation of methodology); Q6 (operator details); Q7 (randomization); Q8 (method of measurement of outcome); Q9 (outcome assessor details); Q10 (blinding); Q11 (statistical analysis); Q12(presentation of results).

### 3.2 Data Synthesis

#### 3.2.1 Bioceramic Sealer × Resin‐Based Sealer

Twelve studies compared the bond strength of fibre posts cemented in root canals filled with bioceramic or resin‐based sealers [12, 13, 14, 15, 16, 17, 18, 19, 20, 21, 22, 38]. Seven studies reported statistically higher bond strength values for resin‐based sealers [12, 13, 14, 15, 16, 17, 18], whereas seven studies found no significant differences between the two groups [15, 16, 17, 19, 20, 21, 22]. One study reported superior results for the bioceramic sealer group [38].

It is worth noting that different outcomes were observed depending on the brand of resin‐based sealer evaluated, the time elapsed between root canal filling and post cementation, as well as the type of resin cement used for post luting. Nesello et al. [17] compared the resin‐based sealer AH Plus with two bioceramic sealers (Bio‐C Sealer and Sealer Plus BC) and observed that when the conventional resin cement RelyX ARC was used, AH Plus produced higher bond strength values than both bioceramic groups. However, when the self‐adhesive cement RelyX U200 was used, AH Plus showed bond strength values similar to those of the bioceramic sealers. Conversely, Soares et al. [15] evaluated Sealer Plus, a resin‐based sealer (not the bioceramic Sealer Plus BC), and reported comparable bond strength values between Sealer Plus and the bioceramic sealer, whereas AH Plus presented significantly higher results than the bioceramic group. Alsubait et al. [16] found higher bond strength for the resin‐based sealer only when the post was cemented immediately after root canal filling, with no significant difference after 7 days. Across all included studies, AH Plus was the most frequently used resin‐based sealer, whereas Sealer Plus appeared only as an additional group in the study by Soares et al. [15].

#### 3.2.2 Bioceramic Sealer × Eugenol‐Based Sealer

Six studies compared the bond strength of fibre posts cemented in root canals filled with bioceramic or eugenol‐based sealers [12, 13, 15, 19, 22, 38]. Three studies reported higher bond strength values for bioceramic sealers [15, 19, 38], while three studies found no significant differences between the two types of sealer [12, 13, 22].

Vilas‐Boas et al. [13] observed similar bond strength values between the sealers when posts were cemented immediately after obturation, but lower values for the bioceramic sealer when post cementation was performed after 1 week.

#### 3.2.3 Resin Based‐Sealer × Eugenol‐Based Sealer

Twenty‐seven studies compared the bond strength of fibre posts cemented in root canals filled with resin‐based or eugenol‐based sealers [7, 8, 10, 12, 13, 15, 19, 22, 26, 27, 28, 29, 30, 31, 32, 33, 34, 35, 36, 37, 38, 39, 40, 41, 42, 43, 44]. Among the studies that used resin‐based sealers, 17 employed AH Plus, 5 used AH26, 3 used Sealer 26, and one study each used EndoRez, Sealer Plus, or TopSeal. Regarding eugenol‐based sealers, 17 studies used Endofill, 2 used Dorifill, and one study each used Pulp Canal Sealer, Sealite, Endomethasone, N‐Rickert, and an unbranded zinc oxide–eugenol sealer.

With respect to bond strength outcomes, 10 studies reported no significant difference between resin‐based and eugenol‐based sealers [8, 10, 22, 26, 28, 29, 35, 41, 42], whereas 19 studies showed significantly higher values for the resin‐based sealers [7, 10, 12, 13, 15, 19, 27, 30, 31, 32, 33, 34, 35, 36, 37, 39, 40, 43, 44]. It is important to highlight that the effect of eugenol was influenced by the time interval between root canal filling and post cementation. For example, Rosa et al. [35] reported that AH Plus presented higher bond strength than Endofill only after 15 days, while no difference was observed when posts were cemented immediately after root canal filling. Similarly, Bohrer et al. [10] found that AH Plus showed significantly higher bond strength than Endofill at 24 h and 6 months, but no significant difference after 12 months.

#### 3.2.4 Adhesive System Type of Resin Cement

Only 15 studies reported the adhesive system used for fibre post cementation. Sixteen studies used self‐adhesive resin cements, including RelyX Unicem, RelyX U100, RelyX U200 (3 M ESPE), and Clearfil SA (Kuraray); 14 studies used conventional resin cements, such as Post Cement Hi‐X (Bisco), Parapost Cement (Coltene), Bistite II DC (J. Morita), Variolink II (Ivoclar), RelyX ARC (3 M ESPE), Duo‐Link (Bisco), AllCem (FGM), and Multilink Automix (Ivoclar); and eight studies used self‐etching resins, including ParaCore (Coltene), Panavia F 2.0 (Kuraray), and Multicore Flow (Ivoclar). Seven studies compared more than one resin cement: five compared self‐adhesive and conventional cements, one evaluated all three categories, and one compared two conventional cements. Across these comparisons, Ruiz et al. [41], Nascimento et al. [42], and Al‐Dwairi et al. [37] reported no differences among adhesive strategies, and all of them tested resin cements in teeth filled with both resin‐based and zinc oxide–eugenol sealers. Regarding bioceramic sealers, Porto et al. [18] found similar bond strength values for Bio‐C Sealer with both RelyX ARC and RelyX U200, whereas Nesello et al. [17] reported higher values for the self‐adhesive cement when associated with Bio‐C Sealer and Sealer Plus BC. For resin‐based sealers, AH Plus showed no difference between self‐adhesive and conventional cements in Nesello et al. [17] and in the cervical third in Porto et al. [18]. In Bohrer et al. [10], the self‐adhesive cement performed better after 24 h, but the conventional cement showed higher bond strength at 6 and 12 months. When Endofill was used, self‐adhesive cements demonstrated higher values at 24 h and 6 months, with similar performance to the conventional cement after 12 months Bohrer et al. [10].

#### 3.2.5 Post Cementation Time

The bond strength evaluation periods varied among the included studies. Most studies assessed bond strength 7 days after root canal filling. Five studies evaluated it after 5 days, and three studies assessed it 24 h after filling. Finally, four distinct studies investigated the least frequent time intervals, as follows: 48 h (one study), 28 days (one study), 12 months (one study), and 30 months (one study).

Only eight studies compared more than one time interval between root canal filling and post cementation. Among the studies that used resin‐based sealers, time did not significantly affect bond strength in Menezes et al. [28], Rosa et al. [35], Vilas‐Boas et al. [13], and Yuanli et al. [21]. Similarly, Bohrer et al. [10] reported that, when using the RelyX U200 resin cement, no statistical difference was observed between the 6 month and 12 month evaluation periods. Conversely, some studies demonstrated an increase in bond strength over time. Bohrer et al. [43] reported significantly higher values after 30 months compared with 24 h, while Ruiz et al. [41] observed greater bond strength after 6 months than after 7 days. In contrast, other studies showed a reduction in bond strength over time. Bohrer et al. [10] reported a decrease between 24 h and 6 months when using RelyX U200, and Alsubait et al. [16] found higher bond strength when post cementation was performed immediately after root canal filling compared with a 1‐week delayed procedure.

Regarding bioceramic sealers, Vilas‐Boas et al. [13] and Alsubait et al. [16] found no significant difference in bond strength between immediate and delayed post cementation after root canal filling. Conversely, Yuanli et al. [21] reported lower bond strength after 1 week compared with immediate cementation, particularly in the cervical and apical root thirds.

Regarding eugenol‐based sealers, Rosa et al. [35] and Vilas‐Boas et al. [13] found no significant difference between immediate and delayed post cementation after root canal filling. Similar results were reported by Bohrer et al. [10], who evaluated the self‐adhesive resin cement RelyX U200 and observed that although a reduction in bond strength occurred between 24 h and 6 months, no significant difference was detected between 6 and 12 months. Conversely, some studies reported an increase in bond strength over time. Menezes et al. [28] recorded higher values when post cementation was performed 7 days after filling compared to the immediate procedure, and Ruiz et al. [41] observed superior bond strength after 6 months compared with the one‐week evaluation. In the opposite direction, Bohrer et al. [43] reported a significant decrease in bond strength in canals filled with an eugenol‐based sealer when posts were cemented after 30 months, with values lower than those recorded at 24 h.

## 4 Discussion

This systematic review evaluated the influence of eugenol‐based, resin‐based and calcium silicate–based endodontic sealers on the bond strength between fibreglass posts and root canal dentine. A total of 33 studies met the inclusion criteria by comparing at least two types of root canal sealers (resin‐based, eugenol‐based, or bioceramic). Likewise, studies evaluating only a single sealer type or using metallic or carbon fibre posts were excluded to ensure methodological consistency [45, 46, 47, 48, 49, 50, 51, 52, 53, 54, 55, 56, 57, 58]. Studies using human and bovine teeth were included, as previous evidence indicates no significant structural differences between the dentine of these species [35, 59]. However, due to the substantial heterogeneity among the included studies, a reliable quantitative synthesis through meta‐analysis was not feasible.

The post cementation time varied among the included studies. Post‐cementation interval is an important variable that may influence bond strength; however, for resin‐based sealers, this effect appears to be clinically negligible. Most studies reported no significant changes in bond strength when posts were cemented immediately, after a few days, or even several months following root canal filling [13, 21, 28, 35]. This finding is expected, as resin‐based sealers achieve a stable and highly polymerised structure shortly after setting, resulting in minimal chemical interaction with resin cements over time [10, 16]. Consequently, delaying post cementation does not appear to compromise adhesion. Although a few studies have reported isolated increases or decreases in bond strength at specific time points, these do not form a consistent trend. Overall, the available evidence indicates that, for resin‐based sealers, the timing of post cementation has minimal impact on bonding performance, supporting both immediate and delayed procedures [13].

Similarly, Vilas‐Boas et al. [13] reported that the timing of post cementation, whether immediate or after 1 week, does not affect bond strength when calcium‐silicate‐based or eugenol‐based sealers are used. Nevertheless, when these findings are compared with those of other studies, the evidence remains inconclusive due to conflicting results regarding whether the cementation interval influences bond strength. A possible explanation for this inconsistency is related to the time‐dependent behavior of eugenol. As described by Bohrer et al. [10], the diffusion of eugenol through dentinal tubules peaks within the first 24 h and then progressively decreases. Because its inhibitory effects on resin polymerization are concentration‐dependent, the reduced diffusion over time may minimize its negative impact on adhesion, which could explain why some studies report no significant differences between immediate and delayed post cementation.

The results appear contradictory regarding the influence of bioceramic sealers on the bond strength when compared with eugenol‐based and resin‐based sealers. Studies comparing bioceramic and eugenol‐based sealers reported either higher bond strength or no significant differences for the bioceramic groups, while comparisons with resin‐based sealers also showed inconsistent outcomes, ranging from superior performance of resin‐based sealers to comparable results, and even isolated findings favoring bioceramic sealers depending on the sealer brand, resin cement used, and timing of post cementation.

The studies that reported a negative influence of bioceramic sealer suggest that the interference in the bond strength of fibre posts is related to the reaction between the calcium hydroxide formed during the hydration process of the sealer and the dentine. This reaction leads to the formation of hydroxyapatite and tag‐like structures that precipitate inside the dentinal tubules, promoting strong adhesion to dentine and making its removal difficult during post space preparation. Consequently, the presence of residual root canal sealer within the dentinal tubules is thought to hinder the interaction of resin cement with the dentine, reducing the bond strength of fibre posts. It has already been demonstrated that the residual percentage of bioceramic sealer in the root canal is higher than that of AH Plus [60], which supports this explanation. Another possible justification is the alkalinity of the residual bioceramic sealer, which may compromise the effectiveness of acid etching and, therefore, the formation of the hybrid layer. However, this remains only a hypothesis proposed by the studies included in this review [13, 14, 15, 17, 21].

When the resin‐based and eugenol‐based sealers were compared, greater bond strength was attributed to resin‐based sealers in most studies. The lower bond strength associated with eugenol‐based sealers is explained by the hydroxyl ions from the phenolic groups in the eugenol molecules, which become trapped in the smear layer of the sealer remnants. These ions bind to the free radicals of the resin monomers, decreasing their reactivity and, consequently, reducing the degree of conversion of the resin, which results in lower bond strength [7, 9]. Some authors advise against the use of eugenol‐containing root canal sealers when cementing fibre posts, regardless of the adhesive system employed with the resin cement [9, 13]. In contrast, epoxy resin–based sealers such as AH Plus do not adversely affect the polymerisation of resin cements. According to Bohrer et al. [10], the superior bond strength observed with AH Plus is attributed to its chemical similarity to resin cements, particularly due to the presence of epoxy resin and the absence of substances capable of interfering with polymerisation, which results in improved interaction at the adhesive interface. Likewise, Alsubait et al. [16] reported that AH Plus and RelyX Unicem share comparable resin‐based compositions, which helps explain the absence of any negative influence of the sealer on the bond strength of fibre posts. Together, these findings support the notion that residual epoxy resin sealer on dentine may even favour adhesion, in contrast to eugenol‐based sealers, which reduce the polymerisation efficiency of resin cements.

Overall, the influence of the adhesive system type on the bond strength of fibre posts remains inconsistent. Most studies found no significant differences among self‐adhesive, conventional, and self‐etching resin cements, regardless of whether the canal was filled with resin‐based or eugenol‐based sealers [37]. When differences were observed, they were not uniform and tended to depend on the sealer type, evaluation period, and root third. For bioceramic sealers, the available evidence is still limited, but current studies suggest that self‐adhesive and conventional cements perform similarly, with occasional superiority of self‐adhesive systems [17, 18]. Taken together, these findings indicate that the choice of adhesive strategy may not be a determining factor in bond strength under most conditions, though more standardised studies, particularly involving bioceramic sealers, are needed to confirm this.

The results from the bias risk assessment revealed significant variation in the methodological quality of the included studies, influenced by the evaluated criteria. Among the 33 studies analysed, most presented a moderate risk of bias, highlighting weaknesses mainly in areas such as detailed sample size calculation, sampling technique, randomization procedures, and blinding of operators and evaluators. These aspects indicate that many studies lack methodological rigour in crucial stages, such as standardisation and impartial analysis of results. In contrast, criteria such as clarity of objectives, detailed explanation of methodology, and presentation of results received higher scores in most studies, indicating that aims and outcomes were generally well described. Only seven studies were classified as having a low risk of bias, reflecting greater methodological robustness, while six studies showed a high risk of bias, suggesting significant shortcomings, especially in randomization, blinding and statistical analysis.

The type of irrigant can influence adhesion. Sodium hypochlorite concentration, in particular, may affect dentine collagen degradation and consequently compromise the adhesion of both endodontic sealers [61, 62] and resin cements [63]. Regarding sodium hypochlorite, fifteen studies irrigated the root canals with 2.5% NaOCl, followed by eight studies that used 5.25% and five studies that used 1%. Additionally, two studies employed 0.5% NaOCl, and one study used a 5% solution. Chlorhexidine, on the other hand, has been shown to inhibit matrix metalloproteinases (MMPs), preventing collagen degradation and improving the longevity of the resin‐dentine bond [64]. However, only one included study used 2% chlorhexidine as an irrigant. These variations in irrigation protocols, both in irrigant type and concentration, may therefore contribute to the heterogeneity observed among the studies.

Another source of variation may be related to the filling technique, as it can influence both the quality of the root canal filling and the ease of material removal during post space preparation [65, 66]. Only four studies performed root canal filling using the warm vertical compaction technique, whereas the remaining studies employed conventional lateral condensation. This inconsistency in filling methods may also contribute to the heterogeneity observed across the included studies.

This review presents important limitations due to the substantial heterogeneity among studies. Differences in root canal diameter, irrigation protocols, obturation techniques, mechanical testing methods, adhesive systems, resin cements, brands of sealers, as well as post‐cementation intervals, all contribute to variability in outcomes and may introduce bias. Furthermore, the limited number of investigations involving bioceramic sealers compared with epoxy‐resin‐based and eugenol‐based sealers represents an additional limitation, underscoring the need for further well‐designed laboratory studies on this material.

## 5 Conclusion

Within the limitations inherent to in vitro studies, resin‐based sealers generally provide higher bond strength values of fibreglass posts cemented with resin cements when compared with eugenol‐based sealers. The influence of eugenol‐based sealers appears to be time‐dependent and remains inconclusive, with studies reporting similar, increased, or reduced bond strength for immediate versus delayed post cementation. Similarly, findings regarding calcium silicate–based sealers are inconsistent when compared with both resin‐based and eugenol‐based sealers, and their effect on fibre post retention remains unclear. The high methodological heterogeneity limits extrapolation to clinical practice, underscoring the need for standardised, long‐term clinical studies.

## Author Contributions

**Thiago Bessa Marconato Antunes** and **Marina Angélica Marciano:** conceptualization, investigation. **Thiago Bessa Marconato Antunes**, **Juliana D. Bronzato, Jennifer Santos Pereira and Vanessa Gallego Arias Pecorari:** methodology, writing – original draft preparation, data curation. **Marina Angélica Marciano**, **Brenda P.F.A. Gomes**, **Thiago Bessa Marconato Antunes**, **Vanessa Gallego Arias Pecorari**, **Adriana de Jesus Soares**, **Talita Tartari, and Juliana D**. **Bronzato:** formal analysis, writing – review and editing All authors commented and corrected the final version of the manuscript. All authors contributed significantly and are in agreement with the manuscript.

## Funding

This study was supported by Fundação de Amparo à Pesquisa do Estado de São Paulo, 2021/11992‐0, 2017/25090‐3, 2021/07920‐4 Coordinação de Aperfeiçoamento de Pessoal de Nível Superior, 001.

## Conflicts of Interest

The authors declare no conflicts of interest.

## Data Availability

The data that support the findings of this study are available on request from the corresponding author. The data are not publicly available due to privacy or ethical restrictions.
